# Evaluation of Antemortem Diagnostic Techniques in Goats Naturally Infected With Scrapie

**DOI:** 10.3389/fvets.2020.517862

**Published:** 2020-11-06

**Authors:** Najiba Mammadova, M. Heather West Greenlee, S. Jo Moore, Soyoun Hwang, Aaron D. Lehmkuhl, Eric M. Nicholson, Justin J. Greenlee

**Affiliations:** ^1^Virus and Prion Research Unit, National Animal Disease Center, Agricultural Research Service, United States Department of Agriculture, Ames, IA, United States; ^2^Department of Biomedical Sciences, Iowa State University College of Veterinary Medicine, Ames, IA, United States; ^3^National Veterinary Services Laboratories (NVSL) Diagnostic Bacteriology and Pathology Laboratory, Animal and Plant Health Inspection Service, United States Department of Agriculture, Ames, IA, United States

**Keywords:** goat scrapie, rectal biopsy, optical coherence tomography, retinal thickness, real-time quaking induced conversion, prion disease

## Abstract

Scrapie is a naturally occurring transmissible spongiform encephalopathy (TSE) that affects sheep and goats. Sheep and goats can be infected with scrapie as lambs or kids *via* contact with the placenta or placental fluids, or from ingestion of prions shed in the environment and/or bodily fluids (e.g., saliva, urine, and feces). Like other TSEs, scrapie is generally not diagnosed before extensive and irreversible brain damage has occurred. Therefore, a reliable method to screen animals may facilitate diagnosis. Additionally, while natural scrapie in sheep has been widely described, naturally acquired goat scrapie is less well-characterized. The purpose of this study was to better understand natural goat scrapie in regard to disease phenotype (i.e., incubation period, clinical signs, neuroanatomical deposition patterns of PrP^Sc^, and molecular profile as detected by Western blot) and to evaluate the efficacy of antemortem tests to detect scrapie-positive animals in a herd of goats. Briefly, 28 scrapie-exposed goats were removed from a farm depopulated due to previous diagnoses of scrapie on the premises and observed daily for 30 months. Over the course of the observation period, antemortem biopsies of recto-anal mucosa-associated lymphoid tissue (RAMALT) were taken and tested using immunohistochemistry and real-time quaking-induced conversion (RT-QuIC), and retinal thickness was measured *in vivo* using optical coherence tomography (OCT). Following the observation period, immunohistochemistry and Western blot were performed to assess neuroanatomical deposition patterns of PrP^Sc^ and molecular profile. Our results demonstrate that antemortem rectal biopsy was 77% effective in identifying goats naturally infected with scrapie and that a positive antemortem rectal biopsy was associated with the presence of clinical signs of neurologic disease and a positive dam status. We report that changes in retinal thickness are not detectable over the course of the observation period in goats naturally infected with scrapie. Finally, our results indicate that the accumulation of PrP^Sc^ in central nervous system (CNS) and non-CNS tissues is consistent with previous reports of scrapie in sheep and goats.

## Introduction

Scrapie is a naturally occurring transmissible spongiform encephalopathy (TSE) that affects sheep and goats. Other naturally occurring TSEs, or prion diseases, include bovine spongiform encephalopathy (BSE), transmissible mink encephalopathy (TME), and chronic wasting disease (CWD) in cervids. Sheep and goats are typically thought to be infected with scrapie as lambs or kids *via* contact with the placenta or placental fluids. However, evidence suggests that both sheep and goats can be infected from ingestion of prions shed in bodily fluids [e.g., milk (1–6), saliva (7), and feces (8)] and contaminated environments (9, 10), such as pastures. Animals that may be shedding the scrapie agent and are asymptomatic are rarely identified until the onset of clinical signs, substantially increasing risk of transmission. This has prompted a significant effort to identify an antemortem diagnostic test to screen for asymptomatic carriers of TSEs, including immunoassaying prions in bodily fluids such as saliva, urine, blood, and cerebrospinal fluids [reviewed in (11)].

In sheep and goats infected with natural scrapie, the disease-associated prion protein (PrP^Sc^) has been detected in the lymphoreticular system (e.g., spleen, lymph nodes, palatine tonsils, gut-associated lymphoid tissues, etc.), prompting the development of biopsy tests for the antemortem diagnosis of scrapie using more accessible tissues, including recto-anal mucosa-associated lymphoid tissue (RAMALT) (12), third eyelid (13), or palatine tonsil (14) that are primarily analyzed after immunohistochemistry (IHC). Several studies have described successfully detecting PrP^Sc^ in rectal mucosa in sheep infected with scrapie (15) and cervids infected with CWD (16–20). In infected goats, RAMALT samples collected in the preclinical stages demonstrate a lower percentage of positives as compared to sheep (21) despite accumulation of PrP^Sc^ in other lymphoid tissues (22). Improved diagnosis of scrapie in preclinical goats may require development of techniques with greater sensitivity than IHC or that utilize non-lymphoid tissues.

Here, we evaluated the efficacy of antemortem assessment of retinal thickness measured using optical coherence tomography (OCT) as a preclinical test to detect asymptomatic goats positive for the classical scrapie agent. OCT has emerged as a readily available and non-invasive approach to obtain high-resolution cross-sectional images of the retina and identify changes in retinal structure associated with disease (23, 24). We have previously reported antemortem changes in retinal function and morphology in TME (25) and BSE (26) inoculated cattle several months prior to the onset of clinical illness. Additionally, we and many others have described accumulation of PrP^Sc^ in retinas of animals infected with TSEs (25–38), further supporting the diagnostic potential of retinal imaging to detect antemortem changes in retinal structure associated with TSEs.

In this study, we evaluated the efficacy of antemortem assessment of retinal thickness measured using OCT and RAMALT biopsies analyzed using IHC and real-time quaking-induced conversion (RT-QuIC) as preclinical tests to detect goats that may be positive for the classical scrapie agent.

## Materials and Methods

### Ethics Statement

The laboratory and animal experiments were conducted in Biosafety Level 2 spaces that were inspected and approved for importing prion agents by the US Department of Agriculture, Animal and Plant Health Inspection Service, Veterinary Services. The studies were done in accordance with the Guide for the Care and Use of Laboratory Animals (Institute of Laboratory Animal Resources, National Academy of Sciences, Washington, DC, USA) and the Guide for the Care and Use of Agricultural Animals in Research and Teaching (Federation of Animal Science Societies, Champaign, IL, USA). The protocols were approved by the Institutional Animal Care and Use Committee at the National Animal Disease Center (protocol number: 2711).

### Animals

In 2014, 71 does and 1 buck were depopulated from a premise in Iowa where a case of clinical scrapie had been previously identified in the herd. Of the 72 goats tested, 10 had evidence of PrP^Sc^ in tonsil, retropharyngeal lymph node (RLN), and/or brainstem at the level of the obex. A subset of does (11) was spared from the initial depopulation because they were nursing kids. These 11 does and 17 kids (any female kids, male kids that were born to a scrapie-positive dam, and male kids born on the same day as any other kids that were born to a positive dam) were acquired by the ARS for the purpose of improving antemortem diagnostic techniques for scrapie. In our study, 28 scrapie-exposed goats were observed daily for 30 months. Over the course of the observation period, serial biopsies of RAMALT were taken before the onset of clinical signs and tested using IHC and RT-QuIC, and retinal thickness was measured *in vivo* using OCT. The following doe/kid pairs are shown in this study: 611 born to P83; 594 and 595 born to B197; 596 and 597 born to P78; 606 born to P91; 598 and 599 born to B186; 607 born to P87; and 609 and 610 born to P85. The 28 goats were housed indoors and observed daily. To determine if any goats had haplotypes associated with resistance to scrapie, all goats were genotyped and compared to GenBank Accession number U67922 caprine PrP sequence. None of the goats in this study possessed any of the haplotypes associated with resistance to scrapie (Supplementary Table 1). At the completion of the observation period (30 months), nine of the 28 goats were determined scrapie positive based on accumulation of pathogenic prion protein (PrP^Sc^) by IHC in the brainstem at the level of the obex, the tonsil, and/or the RLN. Incubation period for classical goat scrapie is reported here as the time from birth to the time when unequivocal signs of clinical disease are present. Incubation period for goats that did not show clinical signs of scrapie is reported here as the time from birth to the end of the observation period (30 months).

### Optical Coherence Tomography

Retinal thickness was measured *in vivo* using OCT. Data from at least two time points and 12 goats (six scrapie-positive and six age-matched scrapie-negative goats) were used for this analysis. Each time point was separated on average by ~70 days. The longest time point difference was 175 days between time points 4 and 5. A Bioptigen SD-OCT (Bioptigen, Durham, NC USA) was used to capture linear B scans (6 mm; 1000 A scans/B scan). Scans were taken from dorsocentral retina. At each time point, 10 measurements/animal of retinal thickness were taken from multiple scan frames (using on-screen calipers) to determine an average thickness measurement for each animal. Data were analyzed using a two-way ANOVA, with a Tukey's multiple comparisons test (*post-hoc*). Prism 6 for Windows (GraphPad Software) was used for statistical analysis.

### Real-Time Quaking-Induced Conversion

RT-QuIC reactions were performed as previously described (39–45) for all goats. Briefly, the reaction buffer was composed of 10 mM phosphate buffer (pH 7.4), 300 mM NaCl, 0.1 mg/ml recombinant bank vole prion protein, 10 μM thioflavin T (ThT), and 1 mM ethylenediaminetetraacetic acid tetrasodium salt (EDTA). Reaction buffer (98 μl) was loaded into a 96-well plate with a clear bottom (Nunc, Thermo Fisher Scientific) and seeded with 2-μl dilutions of either brain homogenate or RAMALT in 0.05% SDS/DPBS. The plate was then incubated at 42°C in a BMG FLUOstar Omega plate reader with alternating cycles of 1 min shaking (700 rpm double orbital) and 1 min rest throughout the incubation. ThT fluorescence measurements (460 nm excitation and 480 nm emission) were taken every 45 min. All reactions for each sample were performed in eight replicates (quadruplicates in two independent RT-QuIC assays). To be considered positive, the ThT fluorescence of at least two replicates out of four reactions must be positive. The predefined positive threshold was calculated as 10 SDs above the mean fluorescence of normal cattle brain homogenates. Previously described criteria were applied for classification of positive samples of RT-QuIC (46, 47).

### Immunohistochemistry

For detection of PrP^Sc^, slides were stained by an automated immunohistochemical method, described previously (28, 48). Briefly, paraffin-embedded sections (4 μm) were rehydrated using xylene, followed by a decreasing ethanol concentration gradient (100%, 90% 70%), and a final wash with diH_2_O. Heat-mediated antigen retrieval was performed using citrate buffer (ScyTek Laboratories, Logan, UT) in an autoclave for 30 min. Slides were exposed to a cocktail of the primary antibodies F89/160.1.5 (49) and F99/97.6.1 (50) each at a concentration of 5 μg/ml. Slides were then stained with an indirect, biotin-free staining system containing an alkaline phosphatase-labeled secondary antibody (*ultra*view Universal Alkaline Phosphatase Red Detection Kit, Ventana Medical Systems, Inc., Tucson, AZ) designed for an automated immunostainer (NexES IHC module, Ventana Medical Systems). Slides were counterstained with Gill's hematoxylin and bluing agent (Ventana Medical Systems) and then cover slipped. Images were captured using a Nikon DS camera on a Nikon Eclipse 50i microscope. Micrographs were created using a commercial photo-editing system (Adobe Photoshop and Adobe Illustrator [CC]; Adobe Systems). Antemortem RAMALT and postmortem regulatory samples (brainstem at the level of the obex, tonsil, and RLN) were tested using only monoclonal antibody F99/97.6.1 as previously described (20).

### Western Blot Analysis

Approximately 0.5 g of brainstem material was analyzed as described previously, with minor modifications (51). Samples were homogenized (10% wt/vol) at 4°C in PBS and digested with proteinase K (PK) for 1 h at 37°C. PK digestion was stopped using pefabloc (Roche, Indianapolis, IN) to a final concentration of 0.1 mg/ml. Approximately 1-mg tissue equivalents of homogenate was loaded onto pre-cast sodium dodecyl sulfate (SDS)-12% polyacrylamide gel electrophoresis (PAGE) gels. SDS-PAGE was performed as described by the manufacturer, and the proteins were transferred from the gel to a PVDF membrane with transfer buffer at 35 V for 45 min. The membranes were blocked with 3% BSA in TBS-T (Tris-Buffered Saline + 0.1% Tween-20) and incubated with monoclonal antibody Sha31 (Cayman Chemical, Ann Arbor, MI) at 0.5 μg/ml for 1 h at room temperature or overnight at 4°C. A secondary biotinylated sheep anti-mouse secondary antibody (GE Healthcare, Buckinghamshire, UK) at 0.05 μg/ml and a streptavidin-horseradish peroxidase (HRP) conjugate (GE Healthcare, Buckinghamshire, UK) were used according to the manufacturer's instructions in conjunction with a chemifluorescent detection system (ECL Plus detection system, GE Healthcare, Buckinghamshire, UK) and imaged using a multimode scanner (GBOX, Synoptics).

## Results

### Antemortem Rectal Biopsy Demonstrated PrP^Sc^ in 100% of Scrapie-Positive Offspring Born to Positive Dams, but in Only 50% of Positive Offspring Born to Negative Dams

To determine if antemortem rectal biopsy was an effective way to identify goats that tested positive for the classical scrapie agent, five serial biopsies of rectal mucosa were taken from all goats over the course of the observation period and submitted to the National Veterinary Services Laboratories (NVSL) to test for PrP^Sc^ accumulation by IHC. Table 1 is representative of goats that either tested positive for the scrapie agent or had a positive dam and tested negative for the scrapie agent. All other goats that tested negative for the scrapie agent were omitted from the table. Of the nine goats that were PrP^Sc^ positive by IHC at the end of the study, seven goats had at least one positive antemortem rectal biopsy (Table 1). Neurologic signs that were consistent with scrapie, including ataxia, tremor, head twitch, and reluctance/inability to rise, were observed in five scrapie-positive goats (Table 1). Of the nine scrapie-positive goats, the scrapie status of the dam was known for eight goats. For one scrapie-positive goat (B186), the scrapie status of the dam was unknown. Within a 30-months observation period, all scrapie-positive goats that were born to scrapie-positive dams (*n* = 4) presented with neurologic clinical signs and were positive by rectal biopsy. The remaining four scrapie-positive goats that were born to scrapie-negative dams did not show clinical signs within the 30-months observation period. In these four goats, all antemortem rectal biopsies were negative by IHC. However, antemortem rectal biopsy tested by RT-QuIC demonstrated PrP^Sc^ in two of these four goats. RT-QuIC is a highly sensitive experimental technique that has been demonstrated to be more sensitive than IHC to detect PrP^Sc^ (52). To determine if rectal biopsies determined negative by IHC would also be negative by RT-QuIC, we retrospectively tested *negative* biopsies from goats that were determined to be *positive* postmortem. For all but three of the biopsies tested for two scrapie-positive goats (biopsy 5 for animal 594, biopsies 3 and 4 for animal p88), RT-QuIC results were the same as those obtained by IHC. That is, samples positive by IHC were positive by RT-QuIC, and most samples that were negative by IHC were negative by RT-QuIC (Table 1). Antemortem rectal biopsy demonstrated PrP^Sc^ in 100% (foure of four animals) of scrapie-positive offspring born to positive dams, but in 50% (two of four animals) of positive offspring born to negative dams only when tested by RT-QuIC. Additionally, we found that the scrapie status of the dam does not predict infection of the offspring. Of the 28 goats observed, the scrapie status of the dam was known for 26 goats (Supplementary Table 1). Of these 26 goats, 8 were scrapie positive and 18 were scrapie negative. Three of the 18 scrapie-negative goats were born to a scrapie-positive dam, and four of the eight scrapie-positive goats were born to a scrapie-negative dam.

**Table 1:** Immunohistochemistry and RT-QuIC of serial RAMALT biopsies.

**Animal ID**	**DOB**	**Dam status**	**Scrapie status**	**Clinical signs**	**Rectal biopsies**					**Necropsy date**	**Age at death (years)**
					**1**	**2**	**3**	**4**	**5**		
					**4/3/14**	**6/24/14**	**11/4/14**	**3/31/15**	**6/23/15**		
					**IHC**	**IHC**	**IHC/RT-QuIC**	**IHC/RT-QuIC**	**IHC/RT-QuIC**		
P87	1/12/11	POS	POS	Head twitch, ataxia	POS	POS	XX	XX	XX	8/5/14	3.6
598	1/14/14	POS	POS	Ataxia, tremor	NT	NT	NEG	POS	POS	1/17/17	3
599	1/14/14	POS	POS	Ataxia, reluctant/unable to rise	NT	NT	NEG	POS	POS	1/3/17	3
O68	9/1/13	POS	POS	Ataxia, tremor	NEG	POS	POS	POS	POS	6/30/16	2.8
B186	12/16/07	-	POS	Ataxia, tremor	POS	POS	POS	POS	XX	4/23/15	7.3
594	1/14/14	NEG	POS	NCS	NT	NT	NEG	NEG	*NEG/POS	6/30/16	2.5
P94	6/12/11	NEG	POS	NCS	NEG	NEG	NEG	NEG	NEG	8/24/16	5.2
P88	5/10/11	NEG	POS	NCS	NEG	NEG	*NEG/POS	*NEG/POS	NEG	8/31/16	5.3
610	1/14/14	NEG	POS	NCS	NT	NT	NEG	NEG	NEG	9/1/16	2.6
606	1/14/14	POS	NEG	NCS	NT	NT	NEG	NEG	NEG	6/2/16	2.4
607	1/14/14	POS	NEG	NCS	NT	NT	NT	NT	NT	6/6/14	0.4
P80	12/1/11	POS	NEG	NCS	NEG	NEG	NEG	NEG	NEG	8/24/16	4.7

Representative table shows a summary of results obtained from antemortem rectal biopsies tested for PrP^Sc^ by immunohistochemistry and RT-QuIC. Table shows 12/28 animals. Five of nine scrapie-positive animals had at least one positive rectal biopsy and clinical signs of neurologic disease (rows shaded dark gray). Four of nine scrapie-positive animals had negative rectal biopsies, no observed clinical signs, and a scrapie-negative dam (rows shaded dim gray). Three dams that were scrapie positive had scrapie-negative kids that showed no clinical signs and had negative rectal biopsies (rows shaded light gray). For animals 594 and P88, analysis of rectal biopsy by RT-QuIC was more sensitive than by immunohistochemistry. For all other animals, a similar result was observed by both IHC and RT-QuIC analysis (*). Animals developed clinical scrapie before this biopsy time point (XX).

*NEG, negative; POS, positive; NT, not tested; NCS, no clinical signs; NT, not tested*.

### Changes in Retinal Thickness Are Not Detectable in Goats Over the Course of Incubation With Scrapie

**Figure 1:**
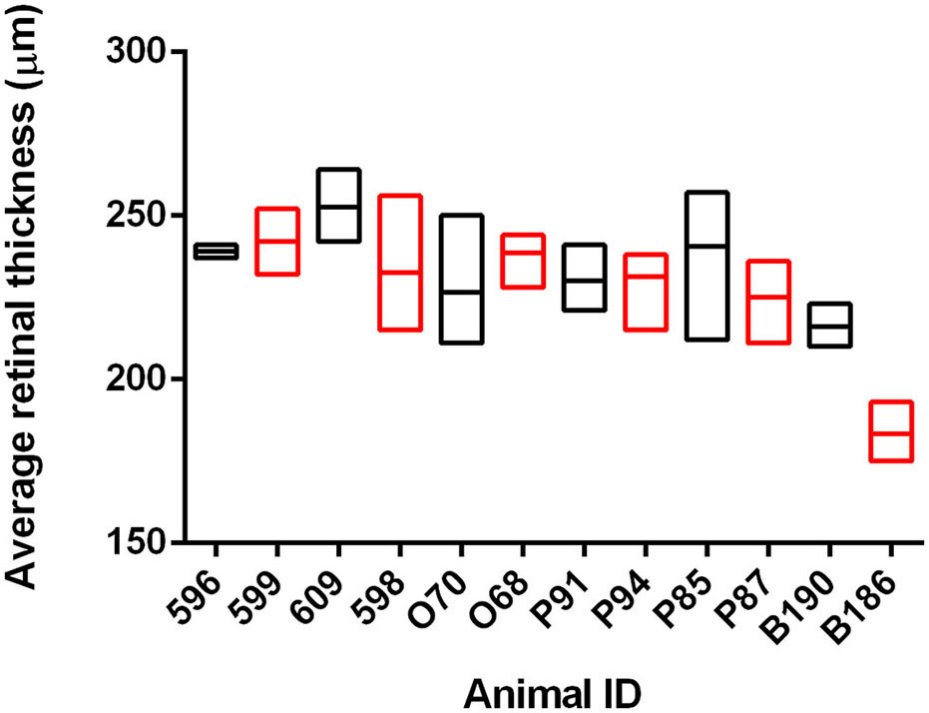
Average retinal thickness (μm) in goats naturally infected with scrapie. Average retinal thickness was measured over time in 12 goats using optical coherence tomography. Floating bars represent average retinal thickness over time (min/max) of scrapie-positive animals (red, *n* = 6 animals/18 measurements) compared to age-matched control animals (black, *n* = 6 animals/21 measurements) from at least two time points. Refer to Supplementary Table 2 for further details.

We have previously shown that OCT can identify changes in retinal thickness in cattle inoculated with BSE up to 11 months prior to clinical signs (26). To determine if there is a similar potential for early detection in goats naturally infected with scrapie, we used OCT to capture retinal images from 12 animals over the course of the incubation period. At the conclusion of the observation period, the average retinal thickness of scrapie-positive animals was compared to age-matched control animals for each time point (Figure 1 and Supplementary Table 2). While the average retinal thickness ± *SD* of scrapie-positive goats (224.9 ± 5.4 μm) was lower than that of scrapie-negative goats (233.1 ± 3.8 μm), there was no statistical significance in retinal thickness between scrapie-positive and scrapie-negative animals at any point during the observation period.

### Accumulation of PrP^Sc^ in Central Nervous System (CNS) and Non-CNS Tissues Is Consistent With Previous Reports of Scrapie in Sheep and Goats

**Figure 2:**
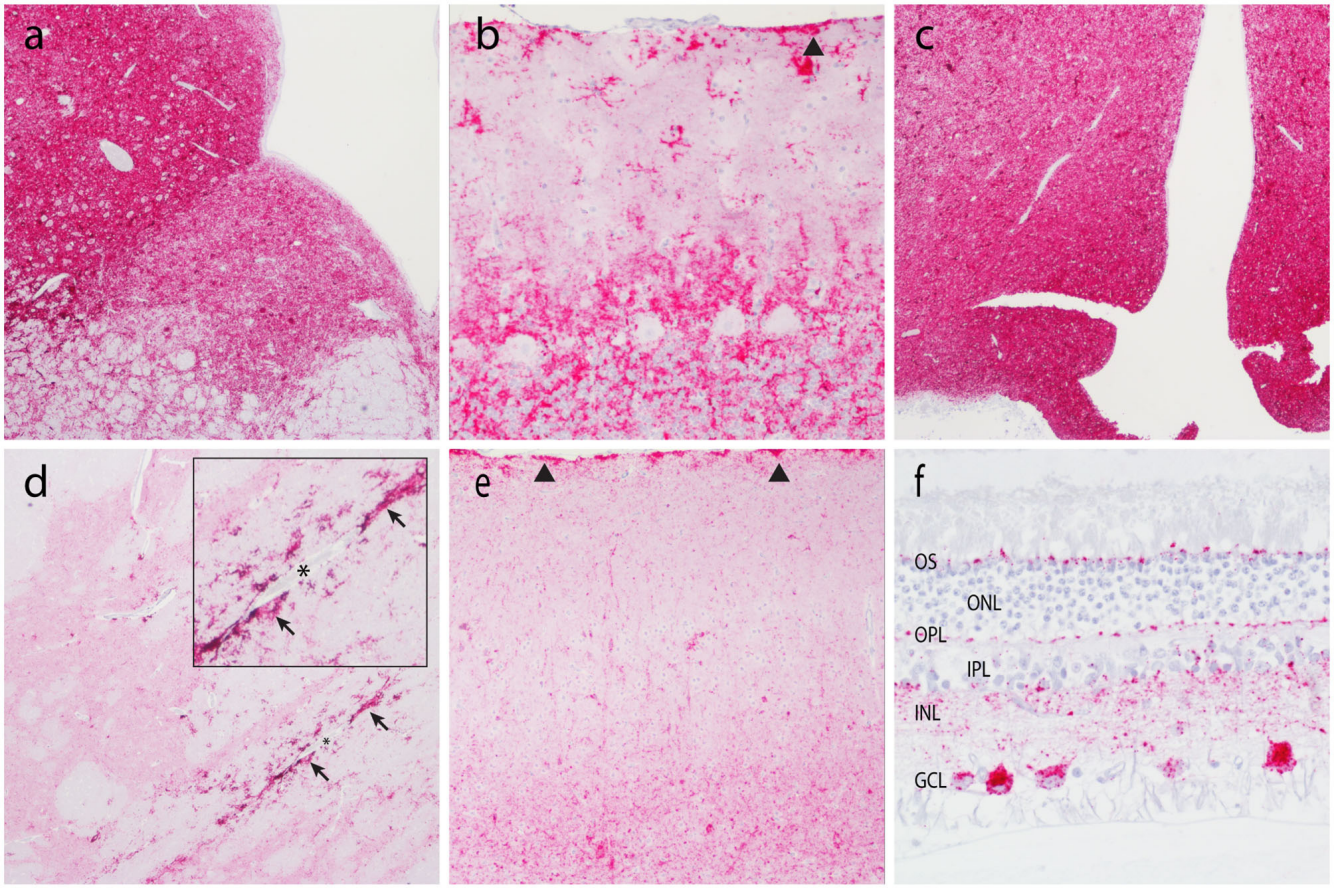
Accumulation of PrP^Sc^ in the central nervous system. Representative micrographs show PrP^Sc^ immunoreactivity in the brainstem at the level of the obex (**a**, 4×), cerebellum (**b**, 20×), thalamus/hypothalamus (**c**, 4×), basal nuclei (**d**, 4×), neocortex (**e**, 10×), and retina (**f**, 40×). Note subpial immunolabeling (arrowheads) in the cerebellum **(b)** and neocortex **(e)**, and prominent perivascular glial-associated immunolabeling (arrows) around a blood vessel (*) in the white matter of the internal capsule **(d)**. Obex, neocortex, and the retina are from animal O68. Cerebellum, internal capsule, and thalamus are from animal B186. OS, outer segments; ONL, outer nuclear layer; OPL, outer plexiform layer; IPL, inner plexiform layer; INL, inner nuclear layer; GCL, ganglion cell layer.

IHC was used to assess PrP^Sc^ accumulation in CNS and non-CNS tissues (Table 2). At the completion of the observation period, nine of the 28 animals were determined scrapie positive based on accumulation of pathogenic prion protein (PrP^Sc^) by IHC in the brainstem at the level of the obex, the tonsil, and/or the RLN. There was no immunolabeling for PrP^Sc^ in any of the scrapie-negative animals. In scrapie-positive animals that presented with clinical signs, PrP^Sc^ immunoreactivity was widespread throughout the brain, spinal cord, and the retina. In these animals, the amount or PrP^Sc^ immunoreactivity was higher in the caudal areas of the brain (brainstem, midbrain, and thalamus) compared to the rostral areas (basal nuclei and neocortex) (Figure 2). In four scrapie-positive animals that *did not* present with clinical signs, PrP^Sc^ immunoreactivity was present in most but not all brain regions. For example, in one animal (594), PrP^Sc^ immunoreactivity was present in more caudal areas of the CNS (obex, medulla, cerebellum, and spinal cord), while absent in more rostral areas (retina, neocortex, basal nuclei, and hippocampus). One animal (P88) had no detectable PrP^Sc^ accumulation in the CNS and was only determined positive based on PrP^Sc^ immunolabeling in the tonsil and RLNs. In another scrapie-positive animal (610), PrP^Sc^ immunoreactivity was only detected in the obex and absent in all other CNS and non-CNS tissues.

**Table 2:** Immunohistochemistry of CNS and non-CNS tissues.

	**Animal ID**								
	**P87**	**598**	**599**	**O68**	**B186**	*594	*P94	*P88	*610
**Incubation period (years)**	3.6	3.0	3.0	2.8	7.3	2.5	5.2	5.3	2.6
**A****ntemortem RB**	POS	POS	POS	POS	POS	POS	NEG	POS	NEG
**Clinical Signs**	+	+	+	+	+	-	-	-	-
**CNS Tissues**									
Neocortex	POS	POS	POS	POS	POS	NEG	POS	NEG	NEG
Basal nuclei	POS	POS	POS	POS	POS	NEG	POS		NEG
Thalamus	POS	POS	POS	POS	POS	POS	POS		NEG
Hippocampus	POS	POS	POS	POS	POS	NEG	POS		NEG
Midbrain	POS	POS	POS	POS	POS	NEG	POS		NEG
Cerebellum	POS	POS	POS	POS	POS	POS	POS		NEG
Medulla	POS	POS	POS	POS	POS	POS	POS		NEG
Obex	POS	POS	POS	POS	POS	POS	POS		POS
Spinal cord	POS	POS	POS	POS	POS	POS	POS		NEG
Retina	POS	POS	POS	POS	POS	NEG	NEG		NEG
**Non-CNS Tissues**									
LRS head	POS	POS	POS	POS	POS	POS	POS	POS	NEG
LRS abdominal/peripheral	POS	POS	POS	POS	POS	POS	POS	POS	
Spleen	POS	POS	POS	POS	POS	POS	NEG	POS	
Lower GIT	POS	POS	POS	POS	POS	POS	NEG	POS	
PNS	POS	POS	POS	POS	POS	POS	NEG	NEG	
Skeletal muscle	NEG	POS	POS	POS	POS	NEG	NEG	NEG	
Adrenal	POS	POS	POS	POS	POS	NEG	NEG	NEG	
Upper GIT	NEG	POS	POS	POS	NEG	NEG	NEG	NEG	
Pituitary	POS	NEG	POS	POS	NEG	POS	NEG	NEG	

*Representative table shows a summary of immunohistochemistry results of CNS and non-CNS tissues only from animals positive for scrapie (n = 9). Gray shading highlights CNS and non-CNS tissues in which PrP^Sc^ was not detected. LRS includes 3rd eyelid, palatine and pharyngeal tonsils, RPLN, MLN, popliteal LN, and prescapular LN. PNS includes optic N., sciatic N., trigeminal G., and dorsal root G. Skeletal muscle includes biceps femoris, masseter, diaphragm, and triceps*.

*RB, rectal biopsy; LRS, lymphoreticular tissues; GIT, gastrointestinal tract; PNS, peripheral nervous system; NT, not tested*.

Experiment was ended before these animals developed clinical signs (*).

**Figure 3:**
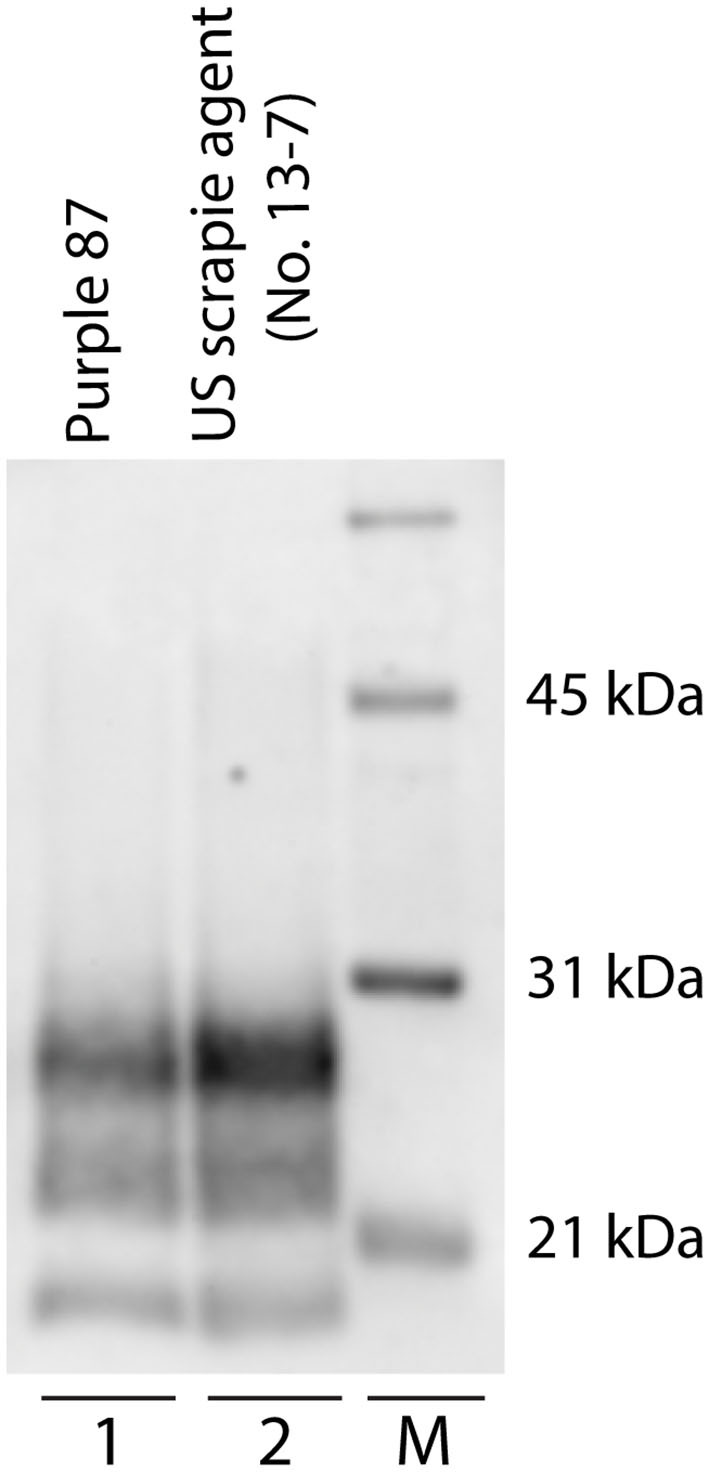
Western blot migration patterns of caprine and ovine scrapie. Proteinase K digestion of brain homogenates from goats and sheep naturally infected with scrapie reveals three immunoreactive bands that represent ratios of three glycoforms. Western blot analysis of PrP^Sc^ reveals similar band patterns of scrapie-positive caprine (lane 1, animal purple 87) and ovine (lane 2). Animal P87 is a representative blot of all scrapie-positive caprine. M: molecular weight marker.

Patterns of PrP^Sc^ immunoreactivity in the brain can be described using standardized morphological immunolabeling types, which are associated with particular cell populations and subcellular locations (53, 54). In goats naturally infected with scrapie, PrP^Sc^ immunoreactivity was present in the neuropil (particulate and aggregated types) and associated with glial cells (stellate, perivascular, and subpial), neurons (intraneuronal, perineuronal, and linear), and microglial cells (intramicroglial). Subpial immunolabeling was observed in the neocortex (Figure 2e) and cerebellum (Figure 2b), and perivascular immunolabeling was prominent in the white matter including the internal capsule at the level of the basal nuclei (Figure 2d). In the retina, particulate PrP^Sc^ immunoreactivity was detected in the outer segments of the photoreceptor cells and the outer and inner plexiform layers, while marked intraneuronal PrP^Sc^ immunoreactivity was present in the ganglion cell layer (Figure 2f). Overall, the patterns of PrP^Sc^ immunoreactivity observed in the brains of scrapie-affected goats in this study are consistent with those reported previously for natural scrapie in sheep and goats [reviewed in (55)]. Additionally, the molecular profile of PrP^Sc^ from brainstem homogenates was analyzed by Western blot for all animals, to compare the migration patterns of caprine and ovine scrapie (Figure 3). Western blot analysis revealed a similar banding pattern between goats and sheep naturally infected with scrapie, shown in a representative blot (Figure 3).

## Discussion

**Figure 4:**
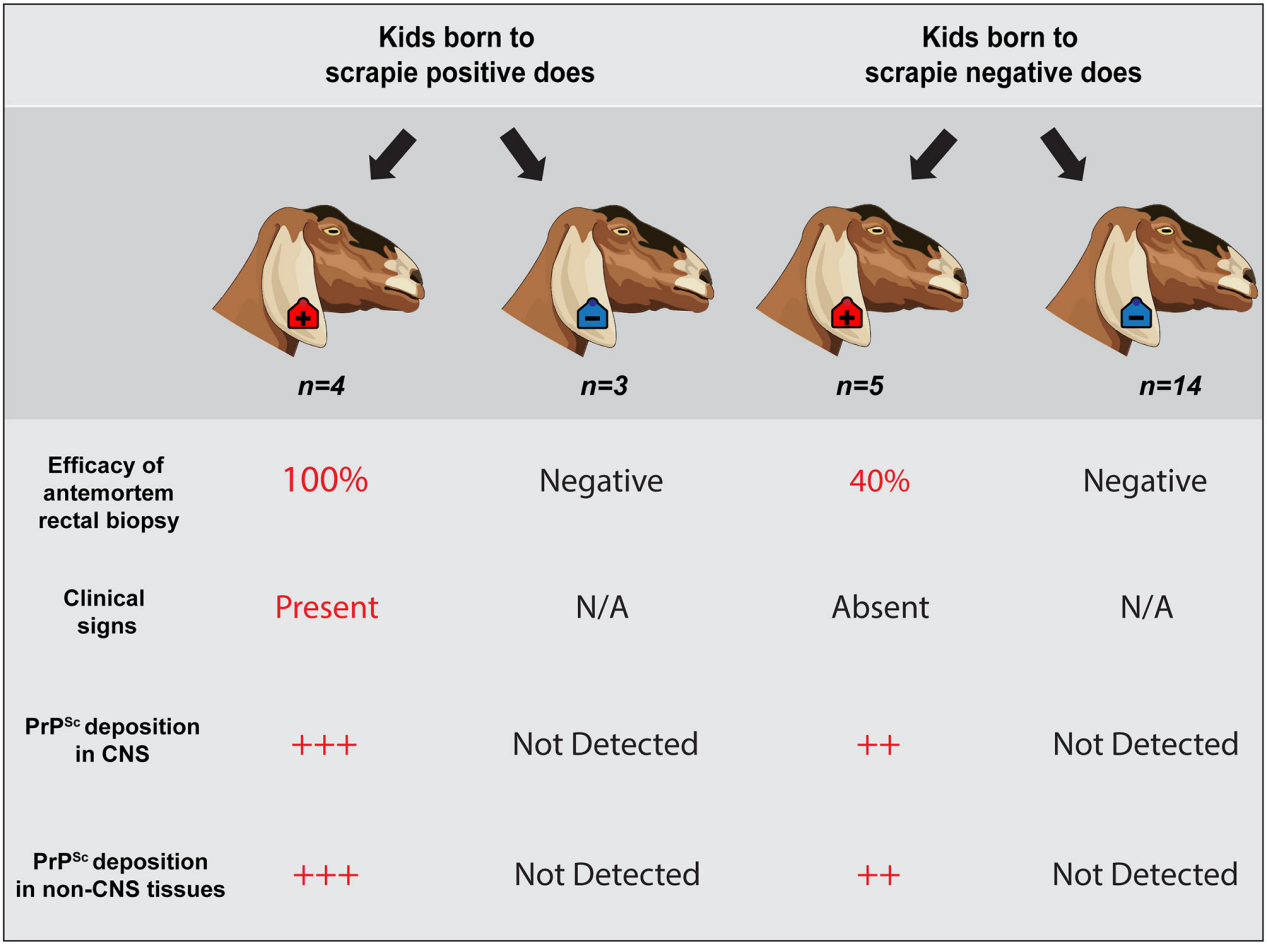
Summary of results. Representative schematic illustrates a correlation between a positive antemortem rectal biopsy, presence of neurologic clinical signs, unequivocal PrP^Sc^ accumulation in *central nervous system and non-CNS tissues*, and a positive dam status. However, scrapie status of the dam does not predict infection of the offspring. Animals observed in this study are highlighted within a dark gray shaded outline (*n* = 26 offspring). Four scrapie-positive goats and 3 scrapie-negative goats were born to seven different scrapie-positive does; five scrapie-positive and 14 scrapie-negative goats were born to 19 different scrapie-negative does. Two goats that were born to does with an unknown dam status are not represented.

In this study, we evaluated the efficacy of antemortem assessment of retinal thickness measured using OCT and IHC and RT-QuIC analysis of RAMALT biopsies as a preclinical test to detect asymptomatic goats positive for the classical scrapie agent. Over the course of a 30-months observation period, serial RAMALT biopsies were taken from asymptomatic goats and tested using IHC and RT-QuIC, and their retinal thickness was measured *in vivo* using OCT. At the completion of the observation period, nine of the 28 goats were determined scrapie positive based on accumulation of PrP^Sc^ by IHC in the brainstem at the level of the obex, the tonsil, and/or the RLN (summarized in Figure 4).

In our study, of the nine goats that were PrP^Sc^ positive by IHC at the end of the observation period, seven goats (77%) had at least one positive antemortem RAMALT biopsy *before the onset of clinical signs*. We found that over the course of a 30-months observation period, a positive antemortem rectal biopsy was associated with the presence of clinical signs and a positive dam status. Three out of these seven scrapie-positive goats (Animal IDs: 598, 599, and O68) had positive antemortem RAMALT biopsies ~21 months before the onset of clinical signs (~58% of the incubation period). Two out of seven scrapie-positive goats had positive antemortem RAMALT biopsies at ~21 and ~23% of their incubation period (Animal IDs: P87 and B186, respectively). Several studies have described detecting PrP^Sc^ in rectal mucosa of cervids that naturally acquired CWD, reporting a relatively high (75–91%) sensitivity of antemortem RAMALT biopsies tested by IHC and/or RT-QuIC (16–18). The efficacy of preclinical diagnosis using RAMALT biopsies has also been reported in sheep infected with scrapie. In a flock known to be scrapie-affected, González et al. reported 30% efficacy of antemortem biopsies of rectal mucosa at 50% of the incubation period and 69% efficacy at 80% of the incubation period (15). Together with our study and others describing low detection levels in positive goats by RAMALT assessment (21), these findings *may* suggest that IHC and/or RT-QuIC analysis of preclinical RAMALT biopsies is a more sensitive method in detecting animals positive for the CWD agent, compared to the scrapie agent. The sensitivity or specificity of RAMALT biopsy as an antemortem diagnostic method is not completely characterized in part because the extent of lymphoid tissue involvement in naturally acquired TSEs can vary considerably, based on several factors (e.g., TSE strain, species, incubation period/age, attack rate, circumstances that the animal acquired the TSE, etc.).

We also examined the comparative efficacy of RAMALT biopsies tested by IHC vs. RT-QuIC, a high-throughput *in vitro* prion amplification system that uses recombinant prion protein as a substrate to amplify otherwise undetectable quantities of PrP^Sc^ seed in tissues and biologic fluids to detectable levels (40, 56–60). Antemortem rectal biopsies determined *negative* by IHC were also retrospectively tested by RT-QuIC (Table 1). Three of 14 (21%) antemortem rectal biopsies tested (2 of 6 goats) were determined negative by IHC and tested positive by RT-QuIC. Few studies have reported a higher sensitivity of RT-QuIC vs. IHC in detecting PrP^Sc^ in rectal mucosa (16, 19) and third eyelid (61) samples of cervids naturally and experimentally infected with CWD. We found that compared to IHC, RT-QuIC analysis of RAMALT biopsies identified two out of four scrapie-positive goats born to negative dams that were not yet showing clinical signs of scrapie, suggesting that RT-QuIC may serve as a useful method to follow preclinical goats over the course of their incubation periods and isolate them prior to the presence of clinical signs.

Our results suggest that scrapie status of the dam does not predict infection of the offspring. That is, four of the nine goats that were determined scrapie positive had a negative dam, while three of the 19 animals that were determined scrapie negative had a positive dam. Vertical transmission of scrapie has been previously demonstrated in lambs that were fed colostrum and/or milk from classical scrapie-infected ewes and goats (2, 5, 62). Other studies provide evidence for prenatal or *in utero* transmission of scrapie in sheep (63–66); however, conclusive evidence for *in utero* transmission is difficult to achieve considering that scrapie is recognized to have multiple infectious routes. Our results indicated that antemortem rectal biopsy demonstrated PrP^Sc^ in 100% of scrapie-positive offspring born to positive dams, but *in only* 50% of positive offspring born to negative dams possibly due to a delayed disease process resulting from a difference in the route of transmission. That is, animals born to a scrapie-negative dam that were later determined scrapie positive were likely infected with scrapie from ingestion of prions shed in the environment as opposed to contact with the placenta or placental fluids *in utero*, resulting in a delayed disease process and longer incubation times. In this case, antemortem rectal biopsy was a significantly less effective method to identify preclinical animals that later tested positive for scrapie. Altogether, although an animal born to a negative dam can become positive, using antemortem RAMALT biopsy to identify these goats was less effective.

In this study, we demonstrate that assessment of retinal thickness by OCT is not a sensitive screening method to identify goats naturally infected with scrapie. Our results showed that while the average retinal thickness of scrapie-positive goats was lower than that of scrapie-negative goats, there was no statistical significance in retinal thickness between scrapie-positive and scrapie-negative animals over the course of the observation period. We and others have reported accumulation of PrP^Sc^ in retinas of TSE-infected animals (25–38) and changes in retinal structure and function (specifically in cattle infected with TME and BSE) before the onset of clinical illness. While the efficacy of OCT as a potential diagnostic method to detect scrapie in goats has not been previously described, there is an emerging interest in the scientific community to gain insight into retinal pathology associated with neurodegenerative processes including TSEs. This is primarily due to the potential for diagnosis using a non-invasive retinal imaging technique, earlier in the incubation period than is currently possible. Based on our observations reported here, OCT was not a sensitive antemortem detection method in a group of goats naturally infected with scrapie.

Here, we used IHC and Western blot to assess the phenotype of natural goat scrapie, specifically the distribution and pattern of PrP^Sc^ accumulation in CNS and non-CNS tissues, and molecular profile as determined by Western blot. While all goats positive for the scrapie agent had accumulation of PrP^Sc^ in CNS and non-CNS tissues, in animals that presented with clinical signs, PrP^Sc^ immunoreactivity was more widespread throughout the CNS and higher in the caudal areas of the brain compared to the rostral areas. In goats that were positive for the scrapie agent but did not present with clinical signs, PrP^Sc^ immunoreactivity was present in most but not all brain regions. Of note was a single goat (ID #610) with immunoreactivity for PrP^Sc^ in the brainstem but no other brain regions or peripheral or lymphoid tissues. While rare, similar results were obtained from a small number of naturally infected sheep of the VRQ/ARR genotype (67) and approximately 12% of naturally infected elk (68). Perhaps in this single goat, PrP^Sc^ initially had access to the brain via innervation of the oral mucosa or tongue (69) rather than a more traditional oral route that would be expected to result in early accumulations of PrP^Sc^ in the lymphoid tissues. Overall, postmortem analysis of scrapie-positive goats revealed that the phenotype of natural goat scrapie was consistent with previous reports of scrapie in sheep and goats [reviewed in (70)].

Here, we describe several facets of natural goat scrapie, specifically efficacy of RAMALT biopsies and OCT as antemortem diagnostic methods, presentation of neurologic clinical signs, and postmortem analysis (i.e., neuroanatomical deposition patterns of PrP^Sc^ and molecular profiles as detected by Western blot). Our results demonstrate that antemortem rectal biopsy was 77% effective in identifying goats naturally infected with scrapie and that a positive antemortem rectal biopsy was associated with clinical signs of neurologic disease and a positive dam status. Our results show that changes in retinal thickness measured by OCT are not detectable over the course of the observation period in goats naturally infected with scrapie. Finally, we report that the accumulation of PrP^Sc^ in CNS and non-CNS tissues is consistent with previous reports of scrapie in sheep and goats. While natural scrapie in sheep has been widely described, naturally acquired goat scrapie is less characterized particularly due to a historically lower prevalence rate (0.2 vs. <0.1%, respectively). While active surveillance and control/eradication programs have decreased the prevalence of sheep scrapie in the United States to 0.006%, the national prevalence of goat scrapie, while decreased from previous years, was estimated to be higher (<0.02%) than the prevalence of sheep scrapie (USDA, unpublished data). TSEs differ in many aspects including antemortem diagnosis. This warrants the continued evaluation and advancement of conventional and experimental diagnostic methods for the ultimate aim of developing an antemortem diagnostic test that is both non-invasive and sensitive enough to detect PrP^Sc^ in readily accessible tissues and/or biological fluids of asymptomatic animals. Overall, our observations aid in characterizing the sensitivity of preclinical TSE detection methods as well as provide a better understanding of natural goat scrapie.

## Data Availability Statement

The raw data supporting the conclusions of this article will be made available by the authors, without undue reservation, to any qualified researcher.

## Ethics Statement

The animal study was reviewed and approved by Institutional Animal Care and Use Committee at the National Animal Disease Center.

## Author Contributions

MW and JG: conceived and designed the experiments. SH, MW, and JG: performed the experiments. NM, MW, SH, SM, AL, and JG: analyzed/interpreted the data. MW, JG, AL, and EN: contributed resources. NM: prepared figures and wrote the manuscript. NM, MW, SH, SM, AL, EN, and JG: reviewed and edited. All authors read and approved the final manuscript.

## Conflict of Interest

The authors declare that the research was conducted in the absence of any commercial or financial relationships that could be construed as a potential conflict of interest.
