# Supplementary material for: Evaluation of Antemortem Diagnostic Techniques in Goats Naturally Infected With Scrapie
Source: Front Vet Sci. 2020 Nov 6;7:517862. doi: 10.3389/fvets.2020.517862 (PMC7677257; doi:10.3389/fvets.2020.517862)
Supplement: Supplementary file 1 [file Data_Sheet_1.docx]

|  |  |  |  |  |  | **Codons** | | | | | |
| --- | --- | --- | --- | --- | --- | --- | --- | --- | --- | --- | --- |
| **Animal ID** | **Dam Status** | **Scrapie Status** | **Clinical Signs** | **127** | **142** | **143** | **146** | **154** | **211** | **222** | **240** |
| **P87** | POS | POS | Head twitch, ataxia | G | I | H | N | R | R | Q | S |
| **598** | POS | POS | Ataxia, tremor | G | I | H | N | R | R | Q | S |
| **599** | POS | POS | Ataxia, reluctant/unable to rise | G | I | H | N | R | R | Q | S |
| **O68** | POS | POS | Ataxia, tremor | G | I | H | N | R | R | Q | S |
| **B186** | - | POS | Ataxia, tremor | G | I | H | N | R | R | Q | S |
| **594** | NEG | POS | NCS | G | I | H | N | R | R | Q | P/S |
| **P94** | NEG | POS | NCS | G | I | H | N | R | R | Q | P |
| **P88** | NEG | POS | NCS | G | I | H | N | R | R | Q | S |
| **610** | NEG | POS | NCS | G | I | H | N | R | R | Q | P/S |
| **606** | POS | NEG | NCS | G | I | H | N | R | R | Q | P/S |
| **607** | POS | NEG | NCS | G | I | H | N | R | R | Q | S |
| **P80** | POS | NEG | NCS | G | I | H | N | R | R | Q | P |
| **B190** | - | NEG | NCS | G | I | H | N | R | R | Q | P/S |
| **B197** | NEG | NEG | NCS | G | I | H | N | R | R | Q | P/S |
| **P85** | NEG | NEG | NCS | G | I | H | N | R | R | Q | P |
| **P91** | NEG | NEG | NCS | G | I | H | N | R | R | Q | S |
| **P83** | NEG | NEG | NCS | G | I | H | N | R | R | Q | P/S |
| **P78** | NEG | NEG | NCS | G | I | H | N | R | R | Q | P |
| **O70** | NEG | NEG | NCS | G | I | H | N | R | R | Q | S |
| **595** | NEG | NEG | NCS | G | I | H | N | R | R | Q | P |
| **596** | NEG | NEG | NCS | G | I | H | N | R | R | Q | P |
| **603** | NEG | NEG | NCS | G | I | H | N | R | R | Q | P |
| **604** | NEG | NEG | NCS | G | I | H | N | R | R | Q | P |
| **605** | NEG | NEG | NCS | G | I | H | N | R | R | Q | P |
| **608** | NEG | NEG | NCS | G | I | H | N | R | R | Q | P |
| **609** | NEG | NEG | NCS | G | I | H | N | R | R | Q | P |
| **611** | NEG | NEG | NCS | G | I | H | N | R | R | Q | P |
| **597** | NEG | NEG | NCS | G | I | H | N | R | R | Q | P/S |

**Supplementary Table 1: Polymorphisms at codons 127, 142, 143, 146, 154, 211, 222 and 240 in natural scrapie affected goats.** Representative table shows *PRNP* gene polymorphisms at codons 127, 142, 143, 146, 154, 211, 222 and 240 in 28 goats. Abbreviations: NEG, negative; POS, positive; NCS, no clinical signs.

|  |  |  | **Average OCT retinal thickness (µM) measurements over 9 time-points** | | | | | | | | |
| --- | --- | --- | --- | --- | --- | --- | --- | --- | --- | --- | --- |
|  |  |  | **4/17/14** | **6/10/14** | **8/15/14** | **10/8/14** | **4/1/15** | **6/23/15** | **9/23/16** | **10/4/16** | **1/26/17** |
| **Animal ID** | **Dam Status** | **Scrapie Status** | **1** | **2** | **3** | **4** | **5** | **6** | **7** | **8** | **9** |
| **596** | NEG | NEG | NT | NT | NT | NT | NT | 237 | NT | NT | 241 |
| **599** | POS | POS | NT | NT | NT | NT | NT | 252 | NT | 232 | XX |
| **609** | NEG | NEG | 264 | NT | 259 | NT | NT | 242 | 245 | XX | XX |
| **598** | POS | POS | NT | NT | NT | NT | NT | 256 | NT | 227 | 215 |
| **O70** | NEG | NEG | 250 | NT | NT | 220 | NT | 225 | 211 | XX | XX |
| **O68** | POS | POS | 244 | NT | NT | 244 | NT | 228 | XX | XX | XX |
| **P91** | NEG | NEG | 221 | 233 | NT | 225 | 241 | XX | XX | XX | XX |
| **P94** | NEG | POS | 238 | 215 | NT | 235 | 237 | XX | XX | XX | XX |
| **P85** | NEG | NEG | 212 | NT | NT | 252 | NT | 241 | 257 | XX | XX |
| **P87** | POS | POS | 228 | 236 | 211 | XX | XX | XX | XX | XX | XX |
| **B190** | - | NEG | 210 | 223 | NT | NT | 215 | NT | XX | XX | XX |
| **B186** | - | POS | 193 | 182 | NT | 175 | NT | XX | XX | XX | XX |

**Supplementary Table 2: Average retinal thickness (µM) measurements over time in goats naturally infected with scrapie.** Representative table shows a schedule of retinal thickness measurements in 12 goats using optical coherence tomography. Each data point is an average of 10 measurements/animal of retinal thickness taken from multiple scan frames (using on-screen calipers). Shading highlights a scrapie positive animal and its age-matched control animal (herd-mate that was determined to be scrapie negative). Abbreviations: NT, not tested; XX, animal developed clinical scrapie and was euthanized before the OCT timepoint.
